# Ecological and morphological characteristics of parasitoids in *Phauda flammans* (Lepidoptera, Zygaenidae)

**DOI:** 10.1051/parasite/2015036

**Published:** 2015-12-10

**Authors:** Xia-Lin Zheng, Jun Li, Li Su, Jun-Yan Liu, Ling-Yu Meng, Min-Yi Lin, Jing Zhang, Wen Lu

**Affiliations:** 1 College of Agriculture, Guangxi University Nanning 530004 Guangxi Zhuang Autonomous Region P.R. China

**Keywords:** Horticultural pests, Defoliator, Biological control, Parasitoids

## Abstract

*Phauda flammans* Walker (Lepidoptera, Zygaenidae) is one of the notorious defoliators on *Ficus* spp. trees. In order to avoid environmental pollution, potential biological control agents for *P. flammans* need to be investigated instead of chemical control. Four species of insect parasitoids were identified from *P. flammans*, including three hymenopteran species (i.e., *Gotra octocinctus*, *Apanteles* sp. and *Eurytoma verticillata*) and one dipteran species (i.e., *Exorista yunnanica*). Parasitoid ratios of *G. octocinctus*, *Apanteles* sp., *Eu. verticillata* and *Ex. yunnanica* were 7.2%, 4.2%, 1.6% and 0.9%. The four species were all larval endoparasitoids of *P. flammans* larvae. Time of cocoon (pupa) to adult, life span, major axis of cocoon and body length of females were all longer compared to males for *G. octocinctus*, *Apanteles* sp. and *Ex. yunnanica*. Based on the parasitoid ratios, the most abundant parasitoid species was *G. octocinctus*.

## Abstract (fr)

*Phauda flammans* Walker (Lepidoptera, Zygaenidae) est l’un des défoliateurs notoires sur *Ficus* spp. Afin d’éviter la pollution de l’environnement, il est nécessaire d’étudier des agents potentiels de lutte biologique contre *P. flammans*, pour remplacer la lutte chimique. Quatre espèces d’insectes parasitoïdes ont été identifiées chez *P. flammans*, dont trois espèces d’hyménoptères (*Gotra octocinctus*, *Apanteles* sp., *Eurytoma verticillata*) et une espèce de diptères (*Exorista yunnanica*). Les ratios parasitoïdes de *G. octocinctus*, *Apanteles* sp., *Eu. verticillata* et *Ex. yunnanica* étaient 7.2 %, 4.2 %, 1.6 % et 0.9 %. Les quatre espèces étaient toutes des endoparasitoïdes larvaires des larves de *P. flammans*. La durée de cocon (nymphe) à adulte, la durée de vie, la longueur de l’axe majeur de cocon et la longueur du corps était plus élevées chez les femelles que pour les mâles pour *G. octocinctus*, *Apanteles* sp. et *Ex. yunnanica*. Sur la base des ratios de parasitoïdes, l’espèce de parasitoïde la plus abondante était *G. octocinctus*.

## Introduction

*Ficus* spp. trees are the main avenue species in the urban landscape of Southeast Asian countries and southern provinces of China. These trees play an important role in maintaining the ecological balance by actively participating in the cycling of nutrients and gases (e.g., carbon dioxide and oxygen) and providing an enormous leaf area for impingement, absorption and accumulation of air pollutants (e.g., industry, construction materials and vehicle emissions) to reduce the pollution level in the urban atmosphere [3]. Clearly, these functions are weakened when leaves of *Ficus* spp. trees are eaten by herbivorous insects. *Phauda flammans* Walker (Lepidoptera: Zygaenidae) is one of the notorious defoliators on *Ficus microcarpa* L. (Urticales: Moraceae) and *F. racemosa* L. (Urticales: Moraceae) in P.R. China [8–10], and on *F. racemosa* in India [12, 13]. *Phauda flammans* has two generations per year in Nanning City, Guangxi Zhuang Autonomous Region, P.R. China. Larval peak of the first and second generations occurred from mid-May to late June and early August to mid-October, respectively. Larvae of the second and third generations overlapped, which could attribute to the longer developmental duration of larvae. This pest overwintered as the pre-pupae larvae and pupae of the second generation and young larvae of the third generation. Only a few individuals could overwinter in up to 10 mm soil depths [8–10]. Conventional insecticides are still the most effective measure against the pest. However, widespread use of chemical insecticides in the control of this pest is no longer acceptable due to insecticide resistance, negative effects on biodiversity and environmental pollution. For example, dimethoate and dipterex were routinely used to kill *P. flammans* larvae by the Landscape department in our city. In recent years, only high concentrations of these insecticides have been able to kill *P. flammans* larvae. However, residues of the two pesticides are often found in the soil [5].

The purpose of this article is to identify possible biological control agents for *P. flammans* occurring on *Ficus* spp. trees. This study investigated species of parasitoid insects using *P. flammans*, along with their ecological and biological characteristics and parasitoid ratios.

## Materials and methods

**Figure 1.**
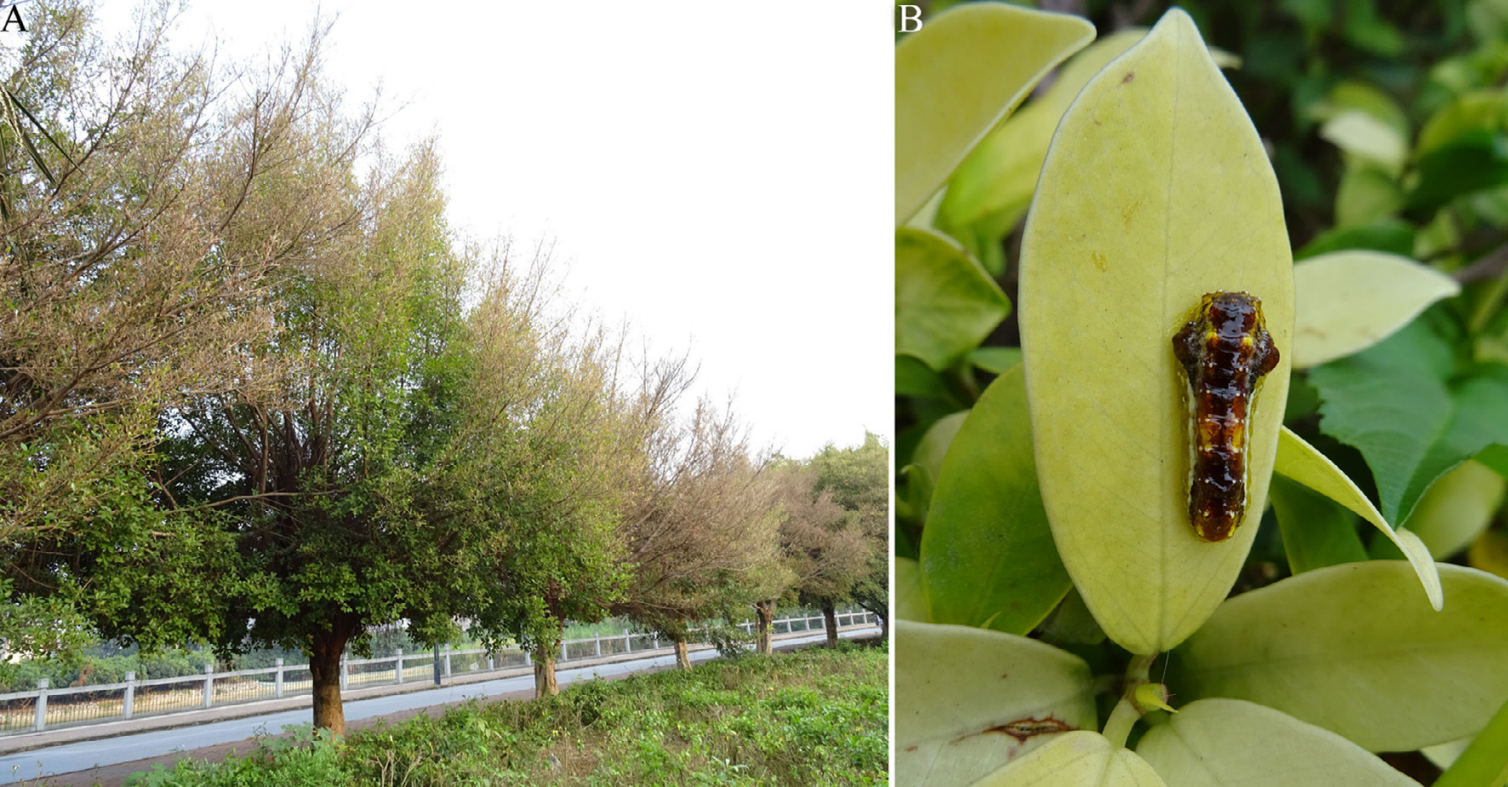
A. *Ficus microcarpa* damaged by *Phauda flammans* larvae; B. *Phauda flammans* larva.

In order to investigate the species of parasitoids, a total of 1032 individuals of *P. flammans* larvae were collected on the damaged trees (Fig. 1) in Nanning City from May to October 2014. Each larva was reared in a petri dish (90 mm diameter × 18 mm height) in a laboratory setting. The collected larvae were incubated at 27 ± 1 °C with an L14: D10 photoperiod and relative humidity (RH) of 70–80%. *Phauda flammans* larvae were reared with leaves of *F. microcarpa* “Golden leaves”. Leaves were renewed and excretions were removed daily from the petri dish until the parasitoids emerged from the host. A total of 25 individuals died during the course of the experiment and no parasitoids were found in their body. A cotton ball soaked in 10% sucrose solution was administered to check the longevity of both sexes of adults if the parasitoids emerged from the host as adults. For parasitoids that emerged from the host as larval stages, cocoon (pupa) were separated (one cocoon/petri dish) after larvae pupated. The sex of each cocoon (pupa) and time of each cocoon (pupa) to adult were recorded, and adults were provided with 10% sucrose solution to record the longevity of both sexes. The major and minor axis of the cocoon (pupa) and body length of adults of these parasitoids in *P. flammans* were measured using image measuring software (Leica Application Suite version 4.6.0, Leica Microsystems, Germany). Rearing conditions of these parasitoids were the same as those of host insects. Images of parasitoid larvae, cocoon (pupae) and/or adults were taken with a Sony digital camera (DSC-HX60, Sony, Kyoto, Japan). All parasitoid adults emerging from the *P. flammans* larvae were identified according to [1, 7, 4, 15].

Statistical analysis was performed using SPSS 16.0 (SPSS, Chicago, IL, USA). Time of cocoon (pupa) to adult, duration of parasitoids in the adult stage, size of parasitoid cocoon and body length of adults of both sexes for each parasitoid were compared using the nonparametric Mann-Whitney *U* test. Results were considered significant at *p* < 0.05.

## Results and discussion

### Species composition of parasitoids and parasitoid ratio

**Figure 2.**
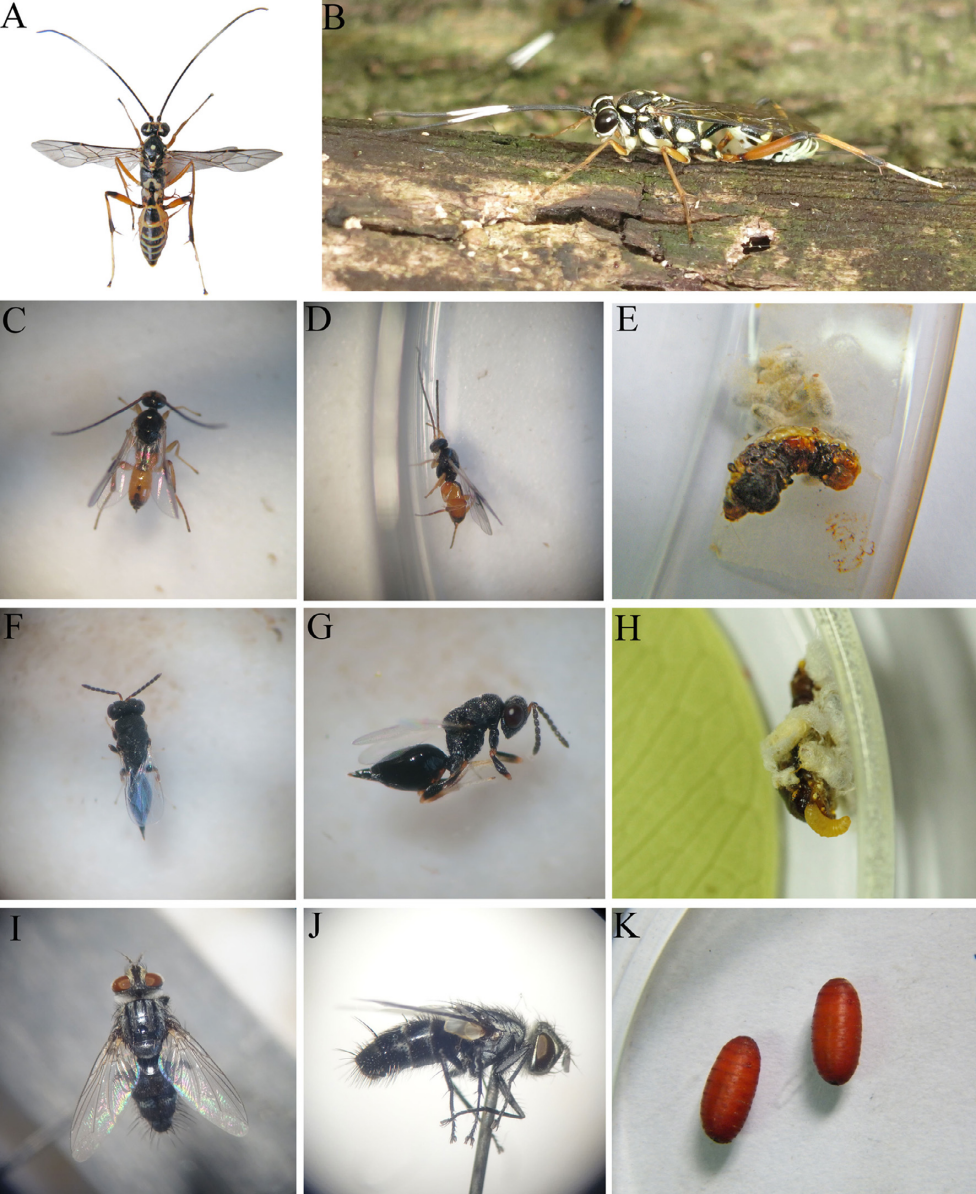
Endoparasitoids of *Phauda flammans* larvae. A–B: Dorsal and profile view of adults in *Gotra octocinctus*. C–E: Dorsal and profile view of adults and cocoon in *Apanteles* sp. F–H: Dorsal and profile view of adults and cocoon in *Eurytoma verticillata*. I–K: Dorsal and profile view of adults and cocoon in *Exorista yunnanica*.

Total parasitoid rate of *P. flammans* larvae was 14.0% (Table 1). The parasitoid ratio of Hymenoptera was 13.1%, which is more than 13 times greater than that of Diptera (0.9%). After rearing the *P. flammans* populations collected from the field, adults of four parasitoid species could be identified: 7.2% *Gotra octocinctus* (Hymenoptera: Ichneumonidae), 4.2% *Apanteles* sp. (Hymenoptera: Braconidae), 1.6% *Eurytoma verticillata* (Hymenoptera: Eurytomidae) and 0.9% *Exorista yunnanica* (Diptera: Tachinidae). Thus, based on the parasitoid ratios, the most abundant parasitoid species was *G. octocinctus* (Fig. 2A).

**Table 1.** Parasitism by parasitoids on *Phauda flammans*.

Order	Family	Species	P.A/C.I	Parasitism (%)		
				Species	Order	Total
Hymenoptera	Ichneumonidae	*Gotra octocinctus*	75/1032	7.2	13.1	14.0
	Braconidae	*Apanteles* sp.	43/1032	4.2		
	Eurytomidae	*Eurytoma verticillata*	17/1032	1.6		
Diptera	Tachinidae	*Exorista yunnanica*	10/1032	0.9	0.9	

C.I, collected individuals from *P. flammans*; P.A, parasitoid appearance in *P. flammans*.

### Ecological characteristics of parasitoids

*Gotra octocinctus* is distributed in Japan, North Korea and P.R. China [4]. Its main hosts are *Dendrolimus punctatus* Walker [14] and *Hyphantria cunea* Drury [6], and the parasitoid ratios were 0.8% and 0.2%, respectively [2, 6]. In this work, we discovered for the first time that *G. octocinctus* also parasitized *P. flammans* larvae in the landscape ecological system of southern cities in P.R. China. This parasitoid emerged from the host as the adult stage (Table 2). Meanwhile, we found only a single parasitoid emerged from a living larva during the process of rearing. This result indicated that this parasitoid was a solitary koinobiont endoparasitoid (Table 2). Competition between parasitoids includes two categories: extrinsic (among free-living adults) and intrinsic competition (among immature parasitoids) [11]. The latter category is usually encountered in solitary parasitoids. Usually, one of the parasitoid larvae will kill the others if two or more solitary parasitoids parasitize the same host. As for *P. flammans*, only a single species of parasitoid *G. octocinctus* finally emerged from the host. We speculate that this may be related to the bigger body size of *G. octocinctus* larvae during interspecific competition among parasitoids in *P. flammans*.

**Table 2.** Ecological characteristics of parasitoids in *Phauda flammans* larvae.

	Parasitized host stage	Parasitoid emerged stage	Parasitized host ecdysis	Food consumption of parasitized host	Solitary (S) or gregarious (G)	Idiobiont (I) or koinobiont (K)
Hymenoptera Ichneumonidae						
* Gotra octocinctus*	Larva	Adult	Yes	Yes	S	K
* *Braconidae						
* Apanteles* sp.	Larva	Larva	Yes	Yes	G	K
* *Eurytomidae						
* Eurytoma verticillata*	Larva	Larva	Yes	Yes	G	K
Diptera						
Tachinidae						
*Exorista yunnanica*	Larva	Larva	Yes	Yes	G	K

*Apanteles* sp. and *Eu. verticillata* were found to parasitize only *P. flammans* larvae. These parasitoids were verified as endoparasitoids and emerged from the host as the larval stage (Table 2). During the experiment, many larvae of *Apanteles* sp. and *Eu. verticillata* were observed to emerge from the host and pupated on the host’s integument (Figs. 2E and 2H). As for *Ex. yunnanica*, they searched for other pupation sites after emerging from the host as the larval stage (Fig. 2K). Results suggested that these species were gregarious. As for the parasitized host, it can exuviate but not pupate which indicated that these parasitoids were koinobiont (Table 2).

It was found that the four species from *P. flammans* larvae were all larval endoparasitoids. Results suggested that the larval stage in the *P. flammans* life cycle was the optimal time used by these parasitoids.

### Morphological characteristics and life cycles of parasitoids

Longevity (*U* = 103.0, *p* = 0.005) and body length (*U* = 115.5, *p* = 0.01) of females were significantly greater than in males in *G. octocinctus* (Table 3).

**Table 3.** Ecological characteristics of *Gotra octocinctus*, *Apanteles* sp., *Eurytoma verticillata* and *Exorista yunnanica*.

	♂/♀	Time of cocoon (pupa) to adult (days)	Duration of parasitoids in adult stage (days)	Size of parasitoid cocoon (cm)		Body length of adults (cm)
				Major axis	Minor axis	
Hymenoptera Ichneumonidae						
* Gotra octocinctus*	♂	–	7.2 (20)	–	–	1.1 (20)
	♀	–	8.0 (21)	–	–	1.2 (21)
* *Braconidae						
* Apanteles* sp.	♂	6.1 ± 0.1 (46)	3.1 ± 0.3 (40)	0.26 ± 0.0 (46)	0.1 ± 0.0 (46)	0.25 (20)
	♀	6.5 (10)	3.6 (10)	0.3 (10)	0.1 (10)	0.29 (10)
* *Eurytomidae						
* Eurytoma verticillata*	♂	6.0 (12)	3.0 (12)	0.3 (12)	0.1 (12)	0.3 (12)
	♀	6.5 (12)	3.3 (12)	0.3 (12)	0.1 (12)	0.3 (12)
Diptera						
* *Tachinidae						
* Exorista yunnanica*	♂	7.0 (8)	4.5 (8)	0.6 (8)	0.4 (8)	0.7 (8)
	♀	7.3 (8)	4.8 (7)	0.7 (7)	0.4 (7)	0.8 (7)

Numbers in parentheses indicate the sample sizes.

With regard to *Apanteles* sp., time for development of cocoon (pupa) of both sexes had a statistical difference (*U* = 145.0, *p* = 0.032). The life spans of adult males and females were 3.1 ± 0.3 and 3.6 days, respectively, which was significantly different (*U* = 118.0, *p* = 0.003). The major and minor axis of male and female cocoon (pupa) were 0.26 ± 0.0/0.1 ± 0.0 cm and 0.3/0.1 cm, respectively, which shows a significant difference of major axis of cocoon (pupa) between males and females (*U* = 2.5, *p* = 0.000) but minor axis was the opposite (*U* = 225.0, *p* = 0.915). Body length of females was significantly longer than males (*U* = 10.0, *p* = 0.000; Table 3), which suggested that the body length could be a positive correlation with the major axis of the cocoon (pupa).

It was found that the period from the cocoon (pupa) to adult of *Eu. verticillata* female was approximately half a day shorter than in males, which does not show a significant difference between the sexes (*U* = 45.0, *p* = 0.085). The life spans of adult males and females were not significantly different (*U* = 58.5, *p* = 0.387). The major and minor axis of male and female cocoon (pupa) were the same as *Apanteles* sp., but there was no statistical difference for males and females (major axis: *U* = 44.0, *p* = 0.105; minor axis: *U* = 56.0, *p* = 0.353). Body lengths of *Eu. verticillata* adults were similar in males and females (*U* = 52.0, *p* = 0.248) (Table 3).

As for *Ex. yunnanica*, the intervals from pupa to adult of males and females were not statistically different (*U* = 25.0, *p* = 0.332). The life spans of adult males and females were 4.5 and 4.8 days, respectively, which does not show a significant difference between the sexes (*U* = 24.0, *p* = 0.317). Although the major axis of female cocoon (pupa) was significantly longer than in males (*U* = 13.5, *p* = 0.031), there was no significant difference of minor axis of cocoon (pupa) between males and females (*U* = 32.0, *p* = 1.000). The body length of females has a significant difference (*U* = 6.5, *p* = 0.007) (Table 3), which suggested that the body length could be a positive correlation with the major axis of the cocoon (pupa). A previous study reported that the body length of *Ex. yunnanica* adults was 7–8 mm [1]. Our results support this conclusion.

## Conclusion

In the current study, we found that *G. octocinctus* was the most abundant species and could be used as a biological agent for *P. flammans* larvae. However, further studies on the mass production and release of this parasitoid need to be carried out.
